# ADAM10 mediates trastuzumab resistance and is correlated with survival in HER2 positive breast cancer

**DOI:** 10.18632/oncotarget.1955

**Published:** 2014-05-08

**Authors:** Katharina Feldinger, Daniele Generali, Gabriela Kramer-Marek, Merel Gijsen, Tzi Bun Ng, Jack Ho Wong, Carla Strina, Mariarosa Cappelletti, Daniele Andreis, Ji-Liang Li, Esther Bridges, Helen Turley, Russell Leek, Ioannis Roxanis, Jacek Capala, Gillian Murphy, Adrian L. Harris, Anthony Kong

**Affiliations:** ^1^ Human Epidermal Growth Factor Group, Department of Oncology, Weatherall Institute of Molecular Medicine, University of Oxford, John Radcliffe Hospital, Oxford, UK; ^2^ Growth Factor Group, Department of Oncology, Weatherall Institute of Molecular Medicine, University of Oxford, John Radcliffe Hospital, Oxford, UK; ^3^ U.O. Multidisciplinare di Patologia Mammaria, U.S Terapia Molecolare e Farmacogenomica, A.O. Instituti Ospitalieri di Cremona, Cremona, Italy; ^4^ National Institutes of Health, Radiation Oncology Branch, Bethesda MD, US; ^5^ School of Biomedical Sciences, Faculty of Medicine, The Chinese University of Hong Kong, Tai Po Road, Sha Tin, Hong Kong; ^6^ Department of Cellular Pathology, Oxford University Hospitals and Oxford Biomedical Research Centre, Oxford, UK; ^7^ Cancer Research UK Cambridge Institute, Li Ka Shing Centre, Robinson Way, Cambridge, UK; ^8^ Institute of Cancer Research, Division of Radiotherapy and Imaging, Belmont, Sutton, Surrey, UK (New address)

**Keywords:** Trastuzumab, resistance, ADAM10, HER2, survival

## Abstract

Trastuzumab prolongs survival in HER2 positive breast cancer patients. However, resistance remains a challenge. We have previously shown that ADAM17 plays a key role in maintaining HER2 phosphorylation during trastuzumab treatment. Beside ADAM17, ADAM10 is the other well characterized ADAM protease responsible for HER ligand shedding. Therefore, we studied the role of ADAM10 in relation to trastuzumab treatment and resistance in HER2 positive breast cancer. ADAM10 expression was assessed in HER2 positive breast cancer cell lines and xenograft mice treated with trastuzumab. Trastuzumab treatment increased ADAM10 levels in HER2 positive breast cancer cells (p≤0.001 in BT474; p≤0.01 in SKBR3) and *in vivo* (p≤0.0001) compared to control, correlating with a decrease in PKB phosphorylation. ADAM10 inhibition or knockdown enhanced trastuzumab response in naïve and trastuzumab resistant breast cancer cells. Trastuzumab monotherapy upregulated ADAM10 (p≤0.05); and higher pre-treatment ADAM10 levels correlated with decreased clinical response (p≤0.05) at day 21 in HER2 positive breast cancer patients undergoing a trastuzumab treatment window study. Higher ADAM10 levels correlated with poorer relapse-free survival (p≤0.01) in a cohort of HER2 positive breast cancer patients. Our studies implicate a role of ADAM10 in acquired resistance to trastuzumab and establish ADAM10 as a therapeutic target and a potential biomarker for HER2 positive breast cancer patients.

## INTRODUCTION

The HER (human epidermal growth factor receptor) family of receptor tyrosine kinases includes four receptors: EGFR, HER2, HER3, and HER4. Several ligands bind to these receptors including heregulin and betacellulin [1]. Ligand binding facilitates homo- or heterodimerization [2], which triggers phosphorylation [3] and activates downstream signalling via the PI3K/AKT and the MAPK pathway [1, 4]. Amplification and overexpression of HER2 occur in about 10-20% of all breast cancer cases, correlating with poorer outcome [5, 6]. Trastuzumab (Herceptin), a humanized monoclonal antibody recognizing HER2 [7, 8], is effective in the treatment of HER2 overexpressing breast cancer in adjuvant and metastatic settings [9-12]. However, the response rate to trastuzumab monotherapy is less than 30% [13] and primary or acquired resistance remains a challenge. Therefore, it is of utmost importance to find strategies to overcome resistance and to establish biomarkers to predict response and resistance to trastuzumab.

Protein shedding via various ADAMs (a disintegrin and metalloproteinase) is important for cell fate determination, migration, and proliferation [14]. ADAM10 has been implicated in the cleavage of a number of proteins such as Ephrins [15], N-cadherin [16], E-cadherin [17], Notch receptor and its ligand Delta 1 [18]. ADAM10 is also involved in the shedding of EGF and betacellulin [19] as well as the HER2 receptor [20]. The role of ADAM10 overexpression was reported in several malignancies such as gastric [21], prostate [22], and liver [23] although its clinical significance in breast cancer is unknown.

We have previously shown that ADAM17 mediates HER receptor activation during trastuzumab treatment [24]. Since ADAM17 and ADAM10 are the two best characterized ADAM proteases responsible for HER ligand shedding [14], we investigated the role of ADAM10 in relation to trastuzumab treatment and resistance in HER2 positive breast cell lines, *in vivo* and in HER2 positive breast cancer patients.

## RESULTS

### Trastuzumab treatment increases ADAM10 levels *in vitro* and *in vivo*

**Figure 1:**
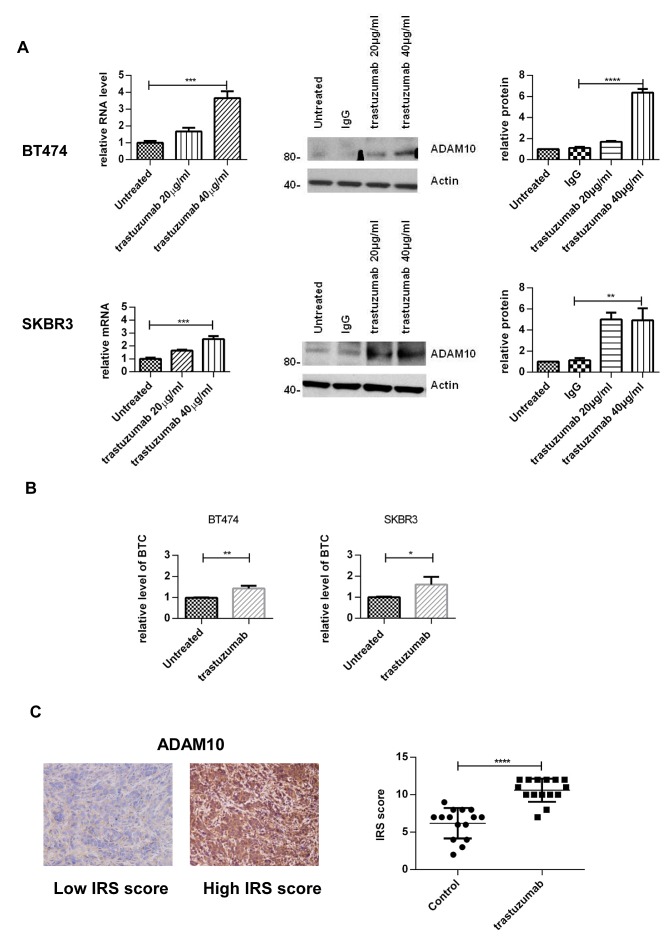
Treatment with trastuzumab leads to an increase of ADAM10 levels *in vitro* and *in vivo* (A) BT474 and SKBR3 cells were treated for 24h with the indicated doses of trastuzumab before ADAM10 mRNA and protein levels were assessed. The relative protein levels from the semi-quantification of three western blots are shown. (B) BT474 and SKBR3 cells were treated with 40μg/ml trastuzumab in serum-free media and betacellulin levels in the media were assessed in triplicate using ELISA after 24h. (C) Paraffin-embedded tumor slides from xenograft mice bearing BT474 tumors treated with trastuzumab (50 mg/kg loading dose and 25 mg/kg maintenance dose administered intraperitoneally twice a week) or saline (control) for a total of 5 doses [30] were stained for ADAM10 expression by IHC before being scored using IRS. Graphs show means ± SD.

To assess the effect of trastuzumab treatment on ADAM10 expression, BT474 and SKBR3 cells were treated with two doses of trastuzumab for 24 hours. ADAM10 mRNA levels were increased in a dose-dependent manner: a 3.6–fold increase in BT474 cells and a 2.5-fold in SKBR3 after 40μg/ml trastuzumab treatment, compared to untreated control (figure 1A, left upper and lower panels, both cell lines, n=3, p≤0.01). ADAM10 protein levels were increased by 7–fold in BT474 and 5–fold in SKBR3 cells (figure 1A, middle and right panels, n=3 each, p≤0.01). The upregulation of ADAM10 coincided with an increase of the ligand betacellulin, which is shed by this protease, in the media of trastuzumab treated cells in comparison to control (figure 1B, n=3, p≤0.05).

To assess the relevance of this observation in an *in vivo* model, tissue samples from BT474 xenograft models treated with trastuzumab or vehicle [30] were stained for ADAM10 expression. The IHC staining was first optimized and was specific (figure S1A). ADAM10 expression was increased in trastuzumab treated xenograft samples (mean IRS 10.6, 95% CI 9.7-11.5) compared to control (mean IRS 6.2, 95% CI 5.0-7.3; n_total_=30, p≤0.0001) (figure 1C). Therefore, trastuzumab treatment led to an upregulation of ADAM10 protein levels *in vitro* and *in vivo*.

### PKB inhibition leads to an upregulation of ADAM10 levels *in vitro* and *in vivo*

**Figure 2:**
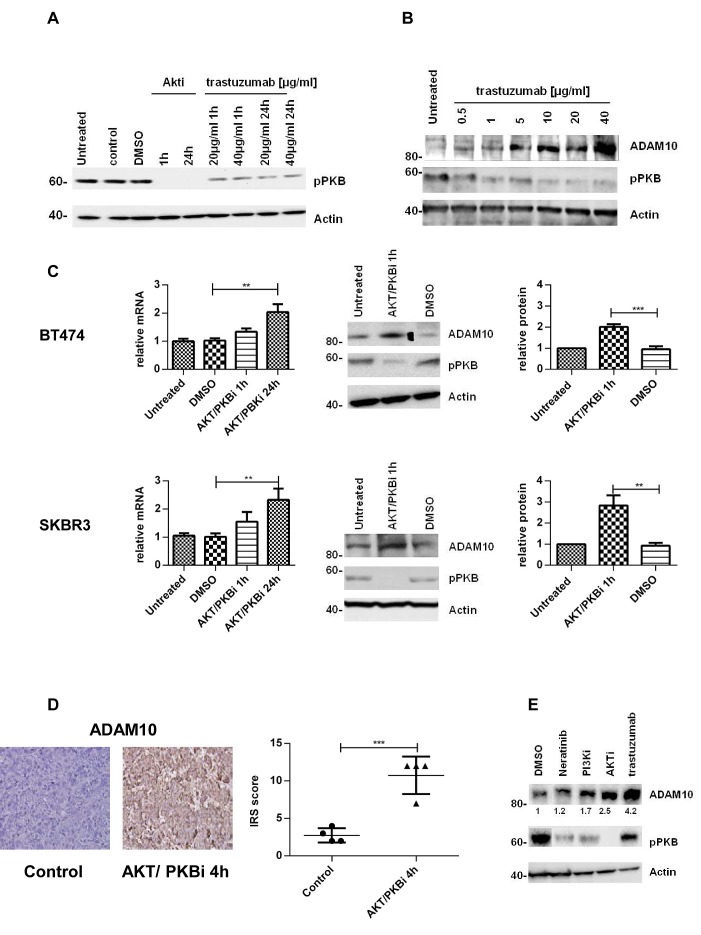
The upregulation of ADAM10 is correlated with AKT inhibition *in vitro* and *in vivo* (A) SKBR3 cells were treated with an allosteric AKT/PKB inhibitor (2.5μM) or trastuzumab for the indicated durations and (B) with increasing doses of trastuzumab for 24h before western blotting. (C) BT474 and SKBR3 cells were treated with an AKT/PKB inhibitor (2.5μM) before ADAM10 mRNA and protein levels were assessed. The relative protein levels from the semi-quantification of three western blots are shown. (D) Paraffin-embedded tumor slides from xenograft mice bearing BT474 tumors treated with an AKT/PKB inhibitor (AKTi-1/2, 50mg/kg ip) or control for 4h were stained for ADAM10 expression by IHC and scored using IRS. (E) SKBR3 cells were treated with 1nM neratinib, 200nM PI3K inhibitor, 2.5μM AKT/PKB inhibitor or 40μg/ml trastuzumab for 24h before western blot. Graphs show means ± SD.

We previously established that the PKB inhibition by trastuzumab [31] increases ADAM17 levels [24]. Thus, we investigated this mechanism in relation to ADAM10. Both trastuzumab and an allosteric AKT/PKB inhibitor could decrease PKB phosphorylation (figure 2A). In a dose escalation study using trastuzumab, the decrease of PKB phosphorylation occurred concomitantly with the increase of ADAM10 protein level. The increase was non-linear although the highest dose of trastuzumab yielded the highest increase of ADAM10 level (figure 2B). To further assess the effect of PKB inhibition on ADAM10 expression, BT474 and SKBR3 cells were treated with the AKT/PKB inhibitor. ADAM10 mRNA level was increased in a time-dependent manner in both cell lines (figure 2C, upper and lower panels, n=3, p≤0.01 at 24h). ADAM10 protein levels were increased at 1h (figure 2C, upper and lower panels, n=3, p≤0.01). The increase was also seen in BT474 xenograft models treated with an AKT/PKB inhibitor (mean IRS 10.8, 95% CI 6.8-14.8) compared to control (mean IRS 2.8, 95% CI 1.2-4.3; n_total_=8, p≤0.001) (figure 2D). We showed that the increase in ADAM10 is related to the decrease in PKB phosphorylation by using other agents targeting this protein (neratinib, a TKI, and a PI3K inhibitor) which also induced ADAM10 protein at 24h of treatment (figure 2E). Collectively, this suggests that PKB inhibition by trastuzumab induces ADAM10 upregulation.

### ADAM10 inhibition decreases basal and trastuzumab induced activation of HER receptors and enhances trastuzumab response

**Figure 3:**
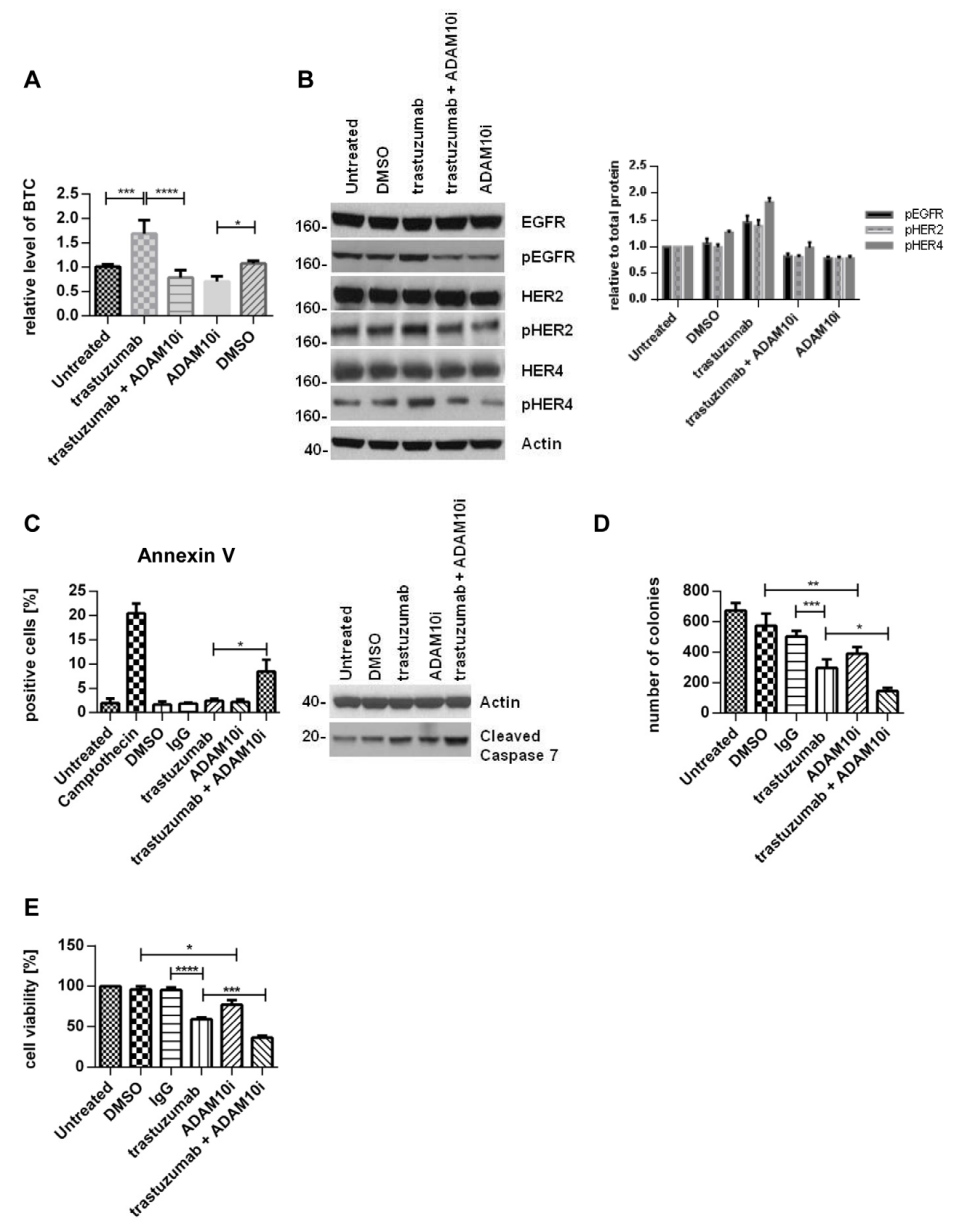
ADAM10 inhibition decreases basal and trastuzumab induced HER activation and enhances trastuzumab response SKBR3 cells were treated with 40μg/ml trastuzumab, 5μM ADAM10 inhibitor INCB8765 (ADAM10i), or their combination in serum-free media as indicated for 24h. (A) Betacellulin levels in the media were assessed in triplicate using ELISA; and (B) cell lysates were used for western blot and quantification of three blots is shown (phosphorylated proteins relative to the respective total proteins). (C) Left, BT474 cells were treated with 40μg/ml trastuzumab, 5μM ADAM10 inhibitor INCB8765 (ADAM10i), or their combination for 5 days in serum-reduced media. Camptothecin (6μM) was used as positive control. Cells were stained for Annexin V and the percentage of positive cells was assessed by FACS analysis. Right, BT474 cells were treated as indicated in serum-reduced media for 72h before western blot. (D) SKBR3 cells were treated as in (C) before cells were re-plated in duplicate and left for colony-formation for 12 days. Cells were then stained and counted. (E) SKBR3 cells were seeded in triplicate and treated as in (C) before MTT assay analysis. Graphs show means ± SD.

The upregulation of ADAM10 by trastuzumab treatment at 24h in both cell lines occurred with an increase of betacellulin (ligand for EGFR and HER4) in the media (figure 3A, n=3, p≤0.001, S1B), correlating with enhanced phosphorylation of EGFR and HER4 receptors and their preferential dimerization partner HER2 (figures 3B left and right panels, S1D).

We therefore proceeded to assess the effect of an ADAM10 inhibitor (ADAM10i) in HER2 positive breast cancer cells. Treatment of SKBR3 and BT474 cells with INCB8765 (specific ADAM10 inhibitor) for 24h led to a decrease in the level of basal (figures 3A, dmso vs. ADAM10i, n=3, p≤0.05, S1B) and trastuzumab induced betacellulin secretion in the media (figures 3A, trastuzumab vs. trastuzumab + ADAM10i, n=3, p≤0.0001, S1B). ADAM10i also decreased cleavage of HER2, another substrate of ADAM10 (figure S1C), as reported previously (20).

In addition, ADAM10i decreased the basal and trastuzumab induced activation of EGFR, HER4, and HER2 in both cell lines (figures 3B, S1C). Although ADAM10 does not shed HER3 ligands (neuregulins), HER3 activation could be affected by dimerization with these receptors. ADAM10i alone did not have much effect on HER3 phosphorylation; however, it was additive to trastuzumab in decreasing HER3 activation (figure S1E). ADAM10i and trastuzumab effects on apoptosis and proliferation were also assessed. Trastuzumab treatment did not enhance apoptosis, as shown by Annexin V staining (figure 3C, left panel), although there was an increased cleaved caspase 7 expression (figures 3C, right panel, S1F) and a decrease in colony formation (figure 3D, IgG vs. trastuzumab, n=3, p≤0.001). However, the combination of trastuzumab with ADAM10i enhanced apoptosis (figures 3C, n=3, p≤0.05, S1F) and inhibited proliferation to a greater extent than trastuzumab treatment alone (figure 3D, n=3, p≤0.05). ADAM10i also decreased cell numbers and enhanced trastuzumab response (figures 3E, n=3, p≤0.05, S1G) in comparison to control. This suggested an additive effect of ADAM10i and trastuzumab.

### ADAM10 knockdown decreases activation of HER receptors but the effect is counteracted by exogenous ligand stimulation

**Figure 4:**
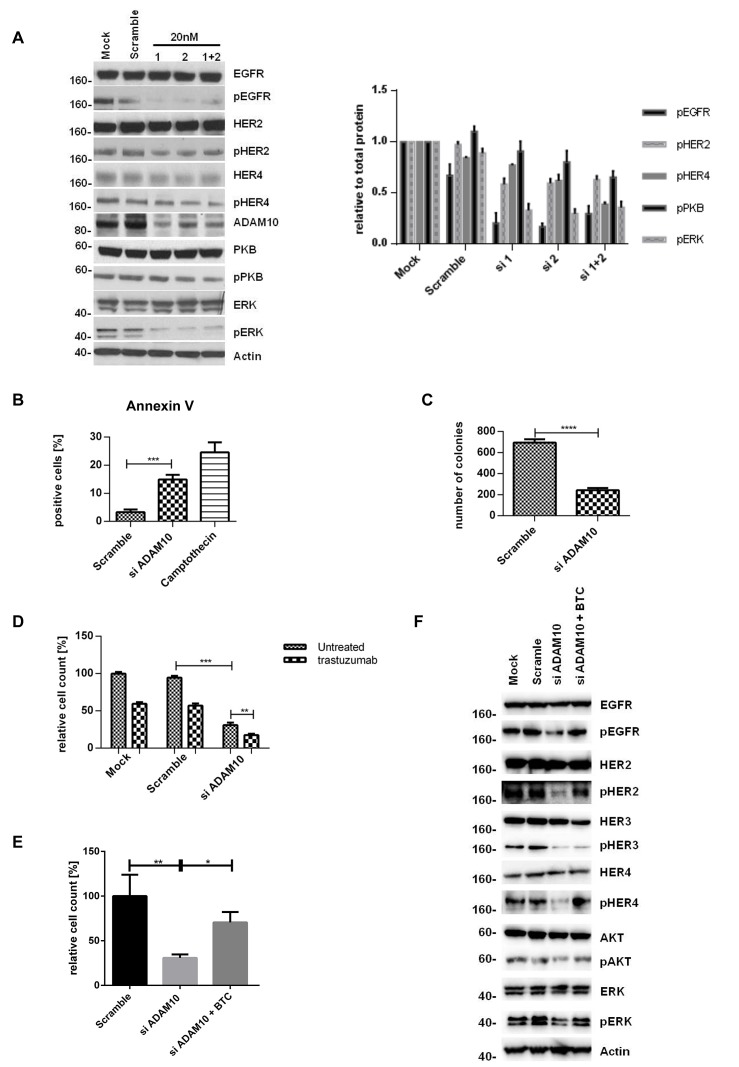
ADAM10 knockdown decreases cell viability and enhances trastuzumab response by inhibiting the activation of HER receptors (A) In SKBR3 cells, ADAM10 was knocked down using two siRNAs (20nM) or their combination before western blotting after 72h. Quantification of three blots is shown (phosphorylated proteins relative to the respective total proteins). (B) BT474 cells were transfected with 20nM of siRNA against ADAM10 for 72h and camptothecin (6μM) was used as positive control. Cells were stained for Annexin V and the percentage of positive cells was assessed by FACS analysis. (C) SKBR3 cells were transfected as in (B) before cells were re-plated in duplicate and left for colony-formation for 12 days. (D) In cell counting assays, SKBR3 cells transfected as in (B) were seeded in triplicate and treated the next day with 40μg/ml trastuzumab as indicated for 5 days. (E) In SKBR3 cells, ADAM10 was knocked down and the cells were co-stimulated with 50ng/ml betacellulin as indicated for 5 days before cell counting experiments (left) or for 72h before western blot analysis for the indicated proteins (F). Graphs show means ± SD.

To further prove the role of ADAM10, we silenced ADAM10 using two different siRNAs (and a combination). The knockdown was first optimized and ADAM17 levels were not affected (figure S2A and B). ADAM10 knockdown decreased the phosphorylation of all HER receptors and the downstream pathways in comparison to control (figures 4A, left and right panels, n=3, S2C, 4E), fortifying the ADAM10 inhibitor results above. It also enhanced apoptosis (figure 4B, n=3, p≤0.001) and decreased colony formation (figure 4C, n=3, p≤0.0001) in comparison to control. Furthermore, ADAM10 knockdown enhanced response to trastuzumab treatment in both cell lines (figures 4D, n=3, p≤0.01, S2D). However, the addition of exogenous betacellulin reversed the inhibitory effect of ADAM10 knockdown on the activation of HER receptors (EGFR, HER2, and HER4) and the downstream pathways, which correlated with an increase in cell proliferation (measured by cell number) compared to ADAM10 knockdown alone (figure 4E). This is not surprising since ADAM10 knockdown effect on the endogenous release of HER ligands and should not have an effect on the exogenous ligand stimulation. HER3 phosphorylation was not recovered by the addition of exogenous betacellulin since it is not a HER3 ligand. These results confirm the role of ADAM10 in the shedding of endogenous HER ligands in HER2 positive breast cancer cells.

### ADAM10 upregulation occurs in acquired trastuzumab resistance and resistant cells are sensitive to ADAM10 inhibition or knockdown

**Figure 5:**
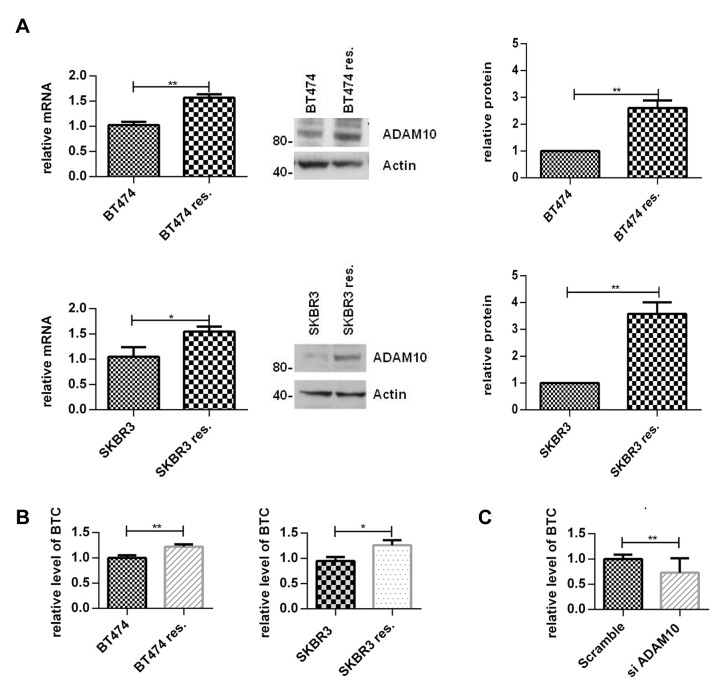
ADAM10 and betacellulin levels are increased in acquired trastuzumab resistant cells compared to naïve cells Resistant cells were continuously treated with 40μg/ml trastuzumab. (A) ADAM10 mRNA and protein levels of naïve and acquired trastuzumab resistant BT474 and SKBR3 cells were assessed. A representative blot and the semi-quantification of three blots are shown. (B) The naïve and acquired trastuzumab resistant BT474 and SKBR3 cells were seeded and the media was replaced the next day by serum-free media for 24h before ELISA. (C) ADAM10 was knocked down in resistant SKBR3 cells and the level of betacellulin in the media was measured by ELISA. Graphs show means ± SD.

**Figure 6:**
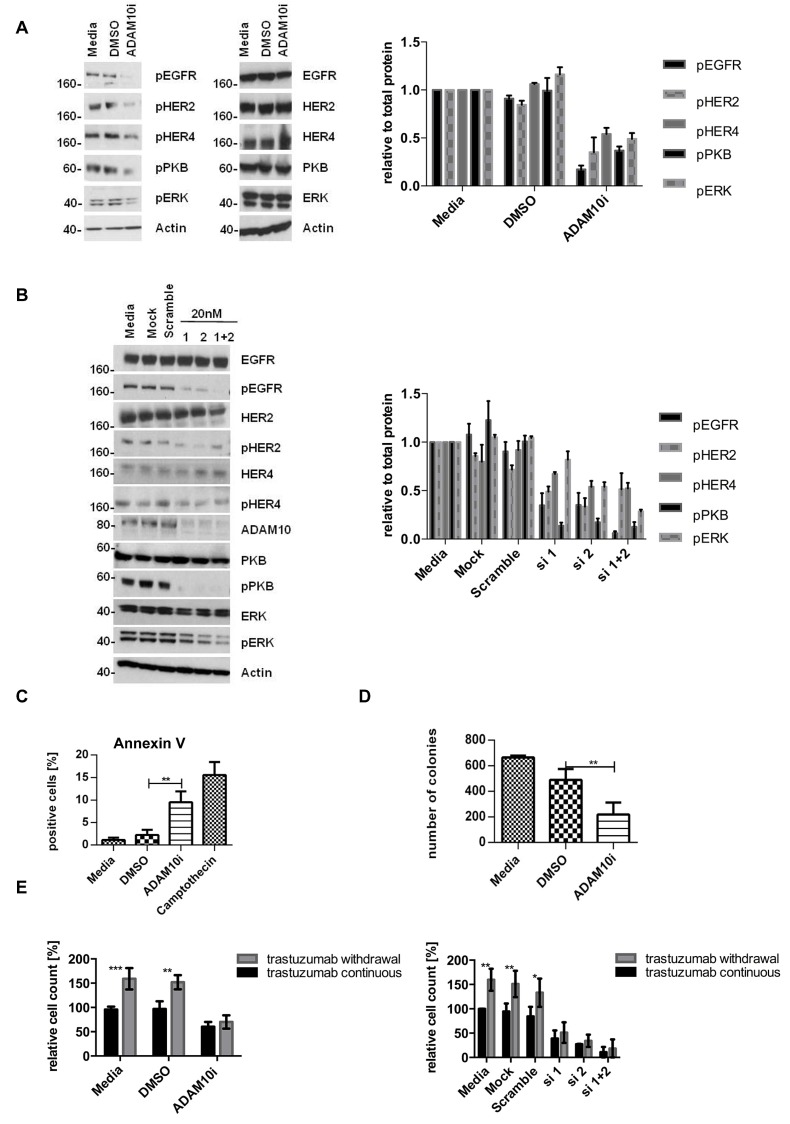

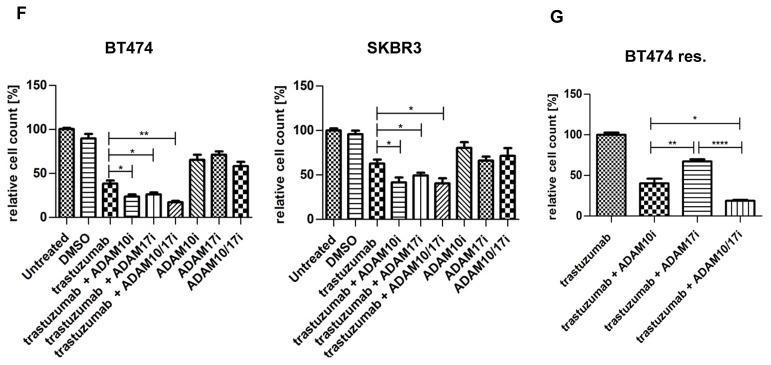
ADAM10 inhibition or knockdown decreases activation of HER receptors and cell viability in trastuzumab resistant cell lines All resistant cells were continuously treated with 40μg/ml trastuzumab unless otherwise stated. (A) Resistant SKBR3 cells were treated with 5μM ADAM10 inhibitor INCB8765 (ADAM10i) for 24h in serum-free media or (B) were transfected with 20nM of siRNAs against ADAM10 for 72h before western blotting and quantification of three blots is shown next to the respective blot (phosphorylated proteins relative to the respective total proteins). (C) For FACS analysis, resistant BT474 cells were treated with 5μM ADAM10 inhibitor INCB8765 (ADAM10i) in serum-reduced media for 5 days. Camptothecin (6μM) was used as positive control. Cells were stained for Annexin V and percentage of positive cells analysed by FACS. (D) For the clonogenic assay, resistant SKBR3 cells were treated as in (C) and re-plated in duplicate for colony-formation for 12 days. (E) For cell counting studies, trastuzumab was withdrawn overnight from resistant SKBR3 cell lines. For the “withdrawal group”, trastuzumab remained withdrawn whereas the “continuous group” was co-treated with 40μg/ml trastuzumab. Both groups were treated in as in (C), or were transfected with 20nM of specific siRNAs against ADAM10 for 5 days (right panel). (F) BT474 and SKBR3 cells were treated in triplicate with 40μg/ml trastuzumab, 5μM of the ADAM10 inhibitor INCB8765 (ADAM10i), the ADAM17 inhibitor INCB4298 (ADAM17i), the ADAM10/17 inhibitor INCB3619 (ADAM10/17i), or their combination for 5 days before cell counting. (G) BT474 resistant cells were treated as in (F) with continuous 40μg/ml trastuzumab for 5 days before cell counting. Graphs represent data from three independent experiments and show means ± SD.

In view of the upregulation of ADAM10 during trastuzumab treatment in naïve cells, we hypothesized that ADAM10 might be implicated in acquired trastuzumab resistance. ADAM10 mRNA and protein levels were increased in the resistant cells compared to parental cells (figure 5A, each n=3, p≤0.05). Moreover, betacellulin levels in the media of resistant cells were also increased (figure 5B, n=3, p≤0.05). ADAM10 knockdown decreased the level of betacellulin in the media of SKBR3 resistant cells (figure 5C). In addition, 24h treatment with ADAM10i (figures 6A; S3A) and ADAM10 knockdown (figures 6B; S3B) decreased HER member phosphorylation and downstream pathway activation in trastuzumab resistant cells. ADAM10 inhibition increased the percentage of apoptotic cells (figure 6C, n=3, p ≤ 0.01) and decreased the number of colonies of trastuzumab resistant cells (figure 6D, n=3, p≤0.01) in comparison to control. However, the effect of ADAM10 knockdown could be counteracted by the addition of exogenous betacellulin in the resistant cells, similar to the result in naïve cells (figure S3C). Although withdrawal of trastuzumab from the resistant cells led to an enhanced proliferation (n=3, p≤0.01), ADAM10 inhibition or knockdown reduced cell numbers compared to control and this effect was independent of trastuzumab treatment in both resistant cell lines (figure 6E; S3D, left and right panels).

### Comparing ADAM10, ADAM17 and ADAM10/17 inhibition in HER2 positive breast cancer cells

We have shown the role of ADAM10 inhibition and knockdown in naïve and resistant HER2 positive breast cancer cells. Previously, we established that ADAM17 inhibition also had an additive effect with trastuzumab treatment [24]. Therefore, we assessed whether a dual inhibition of ADAM10/17 would be superior to inhibition of either ADAM10 or ADAM17 alone. Although all inhibitors enhanced trastuzumab response in SKBR3 and BT474 cells, we found no statistically significant difference in cell viability between the combinations of trastuzumab with either the single inhibitors or the dual one (figure 6F, left and right panels). However, in BT474 resistant cells, ADAM10 inhibition decreased cell count more than ADAM17 inhibition and dual inhibition was superior to either inhibitor alone (figure 6G, n=3, p≤0.05).

### ADAM10 expression correlates with poorer outcome in a HER2 positive cohort

We showed that the upregulation of ADAM10 levels occurred during trastuzumab treatment and upon acquired resistance in HER2 positive breast cancer cells. We hypothesized that ADAM10 expression might be relevant as a biomarker to predict prognosis and trastuzumab response in HER2 positive breast cancer patients. Therefore we stained tumors from HER2 positive breast cancer patients and examples are shown in figure S4A.

**Figure 7:**
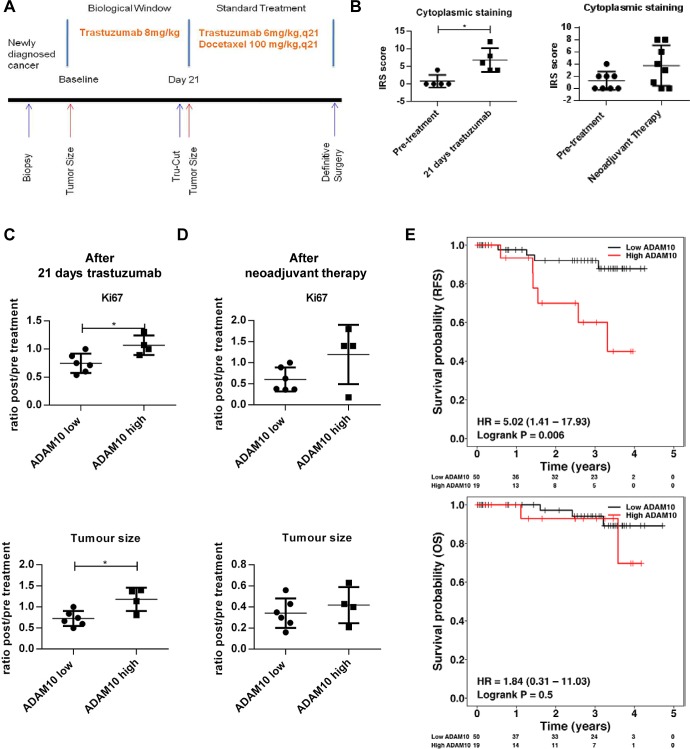
ADAM10 level is a predictive biomarker for trastuzumab response and is prognostic in a cohort of HER2 positive breast cancer patients (A) Schematic illustration of the window of opportunity study. HER2 positive breast cancer patients received a pre-treatment biopsy and underwent a 21 day trastuzumab (8mg/kg) monotherapy window study before a second biopsy was performed. Patients further received 4 cycles of neoadjuvant docetaxel chemotherapy 100 mg/m^2^ with 6mg/kg trastuzumab q21 prior to definitive surgery. (B) Paired tissue samples (pre- and post-treatment) from 5 patients who received trastuzumab monotherapy for 21 days (as described in A) and from 8 patients (of whom samples of pre- and post-neoadjuvant treatment were available) were stained for ADAM10 expression. (C) and (D) Basal ADAM10 expression levels (low or high) of a total of 10 patients (of whom basal biopsies and clinical data were available) were correlated with Ki67 staining and tumor size at day 21 and at definitive surgery (ratios between post trastuzumab or neoadjuvant treatment vs. pre-treatment). Bar graphs show means ± SD, the differences were assessed by t-Test and a p-value is shown (*p≤0.05). (E) Tissue microarrays (TMAs) consisting of tumor core samples from a well annotated HER2 positive breast cancer cohort were stained for basal ADAM10 expression by IHC. Relapse-free survival and overall survival of patients were plotted according to high or low ADAM10 IRS scores and differences between the groups were assessed using Log-rank test.

We assessed ADAM10 levels in patients who underwent a window study as outlined in figure 7A. The characteristics of the patients are listed in table S1. The data from paired samples of patient biopsies (5 out of 10 patients underwent biopsy before and after trastuzumab monotherapy treatment) showed that ADAM10 levels were significantly increased at day 21 after one dose of trastuzumab monotherapy (mean IRS, baseline 0.8, 95% CI -1.4-3.0 vs. post-treatment 6.8, 95% CI 2.6-10.9, p ≤ 0.05) (figure 7B, left panel), similar to the cell lines and xenograft results. Moreover, compared to low basal (= pre-treatment) ADAM10 expression, high basal ADAM10 expression was associated with a higher Ki67 post/pre treatment ratio (high vs. low ADAM expression, mean ratio of 1.1, 95% 0.8 to 1.3 vs. 0.7, 95% CI 0.6-0.9 respectively, p=0.02) and bigger tumor size (high vs. low ADAM10 expression, mean post/pre treatment ratio of 1.2, 95% CI 0.7-1.6 vs. 0.7, 95% CI 0.5-0.9 respectively, p=0.02) at day 21 of trastuzumab monotherapy (figure 7C, upper and lower panels).

We also compared basal ADAM10 levels with those of the tumors obtained at definitive surgery after neoadjuvant chemotherapy and trastuzumab treatment (8 paired samples). There also was an overall trend for increased ADAM10 expression in the post-neoadjuvant treatment samples (figure 7B, right panel). Furthermore, pre-treatment ADAM10 levels also predicted higher Ki67 and poorer clinical response at definitive surgery, although these were not statistically significant (figure 7D upper and lower panels).

To assess the potential prognostic value of ADAM10 levels, we stained a set of consecutive tumor microarrays from a cohort of HER2 positive breast cancer patients for basal ADAM10 expression. Altogether, tumor samples from 69 patients had adequate IHC staining quality to be used for survival analysis. The patient and tumor characteristics are listed in table S2. Higher ADAM10 expression was correlated with a statistically significant poorer relapse-free survival (RFS) compared to low ADAM10 expression (Hazard ratio 5.02, 95% CI 1.41-17.93 p = 0.006). There was also a trend for a lower overall survival although this was not statistically significant (Hazard ratio 1.84, 95% CI 0.31-11.03, p = 0.5) (figure 7E, upper and lower panel). The multivariate analysis using known important clinicopathological factors such as age, node status, tumor grade, ER status and tumor size, suggested that ADAM10 was an independent prognostic indicator for RFS (p=0.0024) (table S3A)

## DISCUSSION

Trastuzumab is a standard treatment for HER2 overexpressing breast and gastric cancer. However, primary or acquired resistance remains a major drawback. This study and our previous study suggested that trastuzumab treatment induced an upregulation of ADAM10 and ADAM17 [24], thereby enhancing ligand release, maintaining HER member activation and counteracting trastuzumab treatment. This was shown to be related to PKB inhibition since several inhibitors (including a TKI and a PI3K inhibitor) that decrease PKB activation induced ADAM10 upregulation. However, the increase in ADAM10 level was not in linear relationship to PKB inhibition. This indicates that there could be several pathways involved in the upregulation of ADAM10, which may include the complex crosstalk between PI3K and MAPK signaling pathways and possible involvement of the FoxO proteins (downstream targets of PKB) [32-34]. This is likely to be complicated and will be investigated further. It will be of interest to assess whether other HER family targeting treatments, such as pertuzumab and lapatinib also induce ADAM10 and 17. HER ligands (e.g. betacellulin and heregulin) shed by ADAM10 and 17, have been shown to mediate resistance to trastuzumab [24] and TKI treatment [35]. HB-EGF and BTC were associated with poor clinical outcome [36] and combined measurement of epigen and neuregulin 4 were predictors of relapse free and overall survival in breast cancer patients [37]. Therefore, it would be important to study further on the interplay between ADAMs with different HER ligands and the activation and dimerization status of HER receptors [38] in the resistance mechanisms of various HER inhibitors in patients.

We showed that naïve and acquired trastuzumab-resistant cell lines were sensitive to ADAM10 inhibition or knockdown through prevention of ligand release and inhibition of HER receptor activation. The effect of ADAM10 inhibition is similar to those seen when inhibiting the tyrosine kinase activities of HER receptors by neratinib [39]. Another possible effect of ADAM10 inhibition is the counteraction of Notch-signalling, which has been implicated in trastuzumab resistance [40] although this is beyond the scope of this manuscript. We showed that the three inhibitors against ADAM10, 17, or 10/17, equally enhanced trastuzumab response in naïve cells but in acquired resistant cells, the dual inhibition was superior. However, all these inhibitors may have non-specific effects on ADAMs and other metalloproteinases [41]. We are currently investigating whether other ADAMs play a role in mediating trastuzumab resistance.

Various studies assessed the clinical significance of ADAMs. ADAM9 was increased in breast cancer in comparison with normal breast tissue and correlated positively with HER2 expression [42]. ADAM17 expression was upregulated in breast cancer compared to normal breast tissue and was highest in lymph-node metastasis [43]. ADAM10 was upregulated in primary liver cancer when compared to normal liver tissue [23]. ADAM10 expression in colon cancer enhanced metastasis [44] and was associated with chemoresistance [45]. Wang et al. (2011) showed that ADAM10 was upregulated in gastric cancer tissue in comparison to normal tissue; higher ADAM10 correlated with poorer prognosis and prognostic markers [21]. Here we report the first study showing the role of ADAM10 in HER2 positive breast cancer. ADAM10 expression did not correlate with any known prognostic markers. However, trastuzumab treatment led to an increase of ADAM10 level in HER2 positive breast cancer patients and higher basal ADAM10 expression was associated with poorer trastuzumab treatment response and relapse-free survival.

Despite the promising results regarding the role of ADAM10 in relation to trastuzumab treatment and resistance shown here, there are limitations to our study. Since we are interested primarily in the role of ADAM10 in acquired trastuzumab resistance, we have not investigated its role in a panel of sensitive and innate trastuzumab resistant breast cell lines. It will be important to assess whether ADAM10 expression could predict response in relation to primary trastuzumab resistance in a wider panel of breast cell lines with different HER2 expression. The biopsy samples used in our window study could also pose problems if there is intra-tumor heterogeneity although the IRS scoring used in this study took into account both the percentage and intensity of ADAM10 staining. The other limitations are the small number of patients used and no statistical plan *a priori* that determined the number of patients in our clinical studies. Thus, despite the statistically significant results shown in our studies, our work is mainly exploratory and provides the hypothesis that ADAM10 expression is a potential prognostic and predictive biomarker. We hope to further validate ADAM10 as a biomarker using samples from randomized controlled trials such as FinHER study [46] in the future.

Our study indicated that ADAM10 is a potential therapeutic target for HER2 positive breast cancers. Friedman et al. reported a phase I/II trial using the compound INCB7839, an ADAM10/17 inhibitor, in combination with trastuzumab [47]. The overall response rate in patients with advanced breast cancer was between 40% and 55% and the new drug was well tolerated [47]. The study also showed that ADAM10/17 inhibitor could reduce HER2 cleavage, which is relevant to HER2 positive patients with tumors expressing p95. We did not focus our study on the role of ADAM10 in HER2 cleavage in patients' samples since there is a lack of validity of p95 assay and there are controversial reports on the role of p95 expression in relation to trastuzumab response and resistance [48, 49]. In view of our data showing the activities of both ADAM10 and ADAM17 in response to trastuzumab, we would support further studies to be done to assess the efficacy of ADAM10/17 inhibitor as a novel therapy to overcome trastuzumab resistance for HER2 positive breast cancer patients. Moreover, the role of ADAM10/17 inhibition in addition to the combination of anti-HER2 therapies such as trastuzumab with lapatinib or pertuzumab should be investigated.

In summary, we showed that an upregulation of ADAM10 level occurred upon trastuzumab treatment and resistance. Inhibition or knockdown of ADAM10 enhanced trastuzumab response in parental cells and reversed acquired trastuzumab resistance in HER2 positive breast cancer cells. For patients who underwent trastuzumab monotherapy, trastuzumab treatment increased ADAM10 expression and pre-treatment high ADAM10 levels predicted poor response to trastuzumab at day 21. Moreover, in a cohort of HER2 positive breast cancer patients, higher basal ADAM10 expressions were correlated with poorer relapse-free survival. Therefore, our results indicate that targeting ADAM10 and ADAM17 might enhance trastuzumab response and overcome acquired trastuzumab resistance in HER2 positive breast cancer patients. We propose further validation studies to confirm ADAM10 level as a prognostic and predictive biomarker in HER2 positive breast cancer patients undergoing trastuzumab treatment.

## MATERIALS AND METHODS

### Cell culture

BT474 and SKBR3 cell lines were provided by cell services lab at Cancer Research UK (Lincoln's Inn Fields laboratory), which has a stringent quality control in cell authenticity and has incorporated short-tandem repeat (STR) profiling for cell line validation. Cell culturing and the generation of trastuzumab resistant cells were described previously [24].

### ADAM10 IHC scoring

ADAM10 expression level was scored semi-quantitatively using immunohistochemistry on tumor samples based on staining intensity and distribution using the immunoreactive score of Remmele and Stegner (IRS) [25]. The scoring criteria is a composite score based on staining intensity (SI) and percentage of positive cells (PP) using the formula IRS = SI × PP. The staining intensity (SI) was determined as 0 = negative; 1 = weak; 2 = moderate; and 3 = strong and the percentage of positive cells (PP) was defined as 0, <1%; 1, 1%–10%; 2, 11%–50%; 3, 51%–80%; and 4, >80% positive cells. Low ADAM10 expression was defined as no or weak staining in tumors (IRS of ≤ 1).

### Human tissue samples and Immunohistochemistry

Tissue microarrays (TMA) were provided by Oxford Radcliffe Biobank in compliance with Human Tissue Act 2004 (UK). The window study of HER2 positive breast cancer patients was carried out as depicted in figure 7A. Tumor size and response were assessed as described [26]. Immunohistochemical evaluation was performed for HER2, ERα, PgR, and Ki67, as described [27]. The trial was conducted at UOM Patologia Mammaria-Az. Instituti Ospitalieri di Cremona with appropriate local ethical approval (Protocol CE-21392/2012). Paraffin-embedded tissues were stained for ADAM10 (Abcam).

### Statistical analysis

Prism 6 software (GraphPad software) was used for statistical analysis. The differences between two means were assessed by t-Test. When investigating one factor, one-way ANOVA was used with Bonferroni's multiple comparison. For two factors, two-way ANOVA with Tukey's multiple comparison [28] was used, specifying the number of comparisons prior to analysis. Associations between the co-variables and ADAM10 level were tested by Fisher's exact test. Overall survival (OS) is defined as percentage of patients still alive in the study period after diagnosis; and relapse-free survival (RFS) is defined as the proportion of patients without relapse (local or distant recurrence) during the defined period [29]. The multivariate Cox proportional hazards modeling and the Kaplan-Meier survival curves analysis were done in R statistical environment (v.2.14.1) (R package: survival v2.36-14). Tests of statistical significance were two-sided and p-values less than 0.05 were considered statistically significant in all the above tests. P values are shown (*p ≤ 0.05, **p ≤ 0.01, ***p ≤ 0.001, ****p ≤ 0.0001).

Further details on materials and methods can be found in the supplementary method section.

## SUPPLEMENTARY METHODS



## SUPPLEMENTAL INFORMATION, FIGURES AND TABLES


