# Supplementary material for: ADAM10 mediates trastuzumab resistance and is correlated with survival in HER2 positive breast cancer
Source: Oncotarget. 2014 May 8;5(16):6633–46. doi: 10.18632/oncotarget.1955 (PMC4196152; doi:10.18632/oncotarget.1955)
Supplement: Supplementary file 1 [file oncotarget-05-6633-s001.pdf]

ADAM10 mediates trastuzumab resistance and is correlated with survival in HER2 positive breast cancer

Supplementary Methods

Western blot

The protocol used for western blotting has been described earlier (1). The primary antibody against ADAM10 was obtained from Calbiochem, and antibodies against pEGFR (Tyr 1173), EGFR, pHER4 (Tyr 1056), and HER4, were purchased from Santa Cruz. All other primary antibodies [HER2, pHER2 (Tyr1221/22), Akt, pAkt (Ser 473), p44/42 MAPK ERK (1/2), p-p44/42 MAPK (Thr 202/Tyr 204) (ERK 1/2), Cleaved Caspase 7 and actin] were from Cell Signaling Technology. Three representative blots were quantified using Image J and values calculated and shown are relative to total proteins where applicable. We used the following compounds or drugs: Trastuzumab (Herceptin, Roche), ADAM10 inhibitor (INCB8765), ADAM17 inhibitor (INCB4298), and ADAM10/17 inhibitor (INCB3619) (all ADAM inhibitors are from Incyte, US), AKT/PKB inhibitor (AKT inhibitor VIII, isozyme-selective, Akti-1/2, Sigma Aldrich), Neratinib (Pfizer) and Wortmannin (PI3K inhibitor, Cell Signaling).

Cell viability and proliferation studies

For MTT assay, cells were treated in triplicate in a 96-well plate. At the end of treatment, MTT solution (USB Corp.) was added and the intensity of colour was measured by a plate reader. For cell counting, cells were treated in 24-well plates in triplicate and afterwards trypsinised and counted. For Annexin V staining, camptothecin (6 µM) (Sigma Aldrich) was used as positive control. Samples were stained with Annexin V (BD Pharmingen) according to manufacturer's instructions and analyzed using FACS. Clonogenic assays were performed as advised by Franken et al. (2006) (2). Briefly, cells were pre-treated for 5 days before re-plating 1000 cells in duplicate in full medium. Colonies were stained with 0.2% methylene blue (Sigma Aldrich) in 50% ethanol.

Quantification of betacellulin levels with ELISA

Betacellulin levels of concentrated media samples were measured using Dual Set ELISA kit (R&D Systems) according to manufacturer's instructions. Briefly, a 96-well plate was coated with a capture antibody. After washing and blocking steps, standards or normalized samples were loaded. This was followed by the addition of a detection antibody, HRP, and a substrate solution. The absorption was measured by a plate reader.

Transient transfection

ADAM10 was knocked down by reverse transfection using oligofectamine (Invitrogen) and ADAM10 specific siRNA or scramble (Sigma Aldrich). Cells were incubated for 48h to assess mRNA levels; for 72h to do western blot analysis; and for 5 days to investigate the effect of knockdown on cell number.

qRT-PCR

RNA was extracted using the RNeasy Mini Kit (Qiagen) according to the manufacturer's instructions. Samples were treated with DNase I (Bio-Rad Lab.). The High Capacity cDNA Reverse Transcription Kit (Applied Biosystems) was used for cDNA synthesis.

For qRT-PCR, 100ng of cDNA was mixed with SensiMixTM SYBER-Hi-ROX qPCR master mix (Bioline Reagents, UK) in triplicate. The following primers were used (Invitrogen): ADAM10 5′-ATATTACGGAACACGAGAAGCTG-3′ and 5'-TCAATCGCTTTAACATGACTGG-3', ADAM17 5'-CCTTTCTGCGAGAGGGAAC-3' and 5'-CACCTTGCAGGAGTTGTCAG-3', Actin 5'-AAT GGC AAT GAG CGG TTC-3' and 5'-GGA TGC CAC AGG ACT CCA T-3'.

Gene expression levels were normalized relative to actin.

Immunohistochemistry staining

After re-hydration, citrate buffer (pH6) was used for antigen retrieval. The ADAM10 antibody (Abcam) was applied overnight at 4°C. Slides were washed with PBS before adding the secondary antibody (ImmPress™ Reagent Anti-Rabbit IgG, Vector Laboratories Inc.). DAB Peroxidase substrate) was added and slides were counterstained with hematoxylin QS (Vector Laboratories Inc.) Slides were scored using the immunoreactive score of Remmele and Stegner (IRS) (3).

Xenograft studies

Xenograft experiments comparing trastuzumab treatment with the vehicle control were described earlier (4). Animal experiments, using an allosteric AKT/PKB inhibitor (AKTi), were carried out independently in Oxford (project license PPL 30/2771) and at the Chinese University of Hong Kong (CUHK) with strict adherence to the guidelines of Guide for the Care and Use of Laboratory Animal (2010 new version, 8th edition) and in accordance with regulations laid down by the Animal Experimentation Ethics Committee (AEEC) of CUHK. BT474 cells were subcutaneously injected into the flank of the NMRI Nude mice, 6-8 weeks old (Charles River Laboratories, Inc. UK). Once the tumors were formed, the mice were treated either with AKTi-1/2 50mg/kg or vehicle (181µl, one injection, intraperitoneally), for 4 hours. Tumor samples were collected and fixed in formalin.

References

1. Gijsen M, King P, Perera T, Parker PJ, Harris AL, Larijani B, et al. HER2 phosphorylation is maintained by a PKB negative feedback loop in response to anti-HER2 herceptin in breast cancer. PLoS Biol. 2010;8:e1000563.
2. Franken NA, Rodermond HM, Stap J, Haveman J, van Bree C. Clonogenic assay of cells in vitro. Nat Protoc. 2006;1:2315-9.
3. Kaemmerer D, Peter L, Lupp A, Schulz S, Sanger J, Baum RP, et al. Comparing of IRS and Her2 as immunohistochemical scoring schemes in gastroenteropancreatic neuroendocrine tumors. Int J Clin Exp Patho. 2012;5:187-94.
4. Kramer-Marek G, Gijsen M, Kiesewetter DO, Bennett R, Roxanis I, Zielinski R, et al. Potential of PET to predict the response to trastuzumab treatment in an ErbB2-positive human xenograft tumor model. J Nucl Med. 2012;53:629-37.
