# Supplementary material for: ADAM10 mediates trastuzumab resistance and is correlated with survival in HER2 positive breast cancer
Source: Oncotarget. 2014 May 8;5(16):6633–46. doi: 10.18632/oncotarget.1955 (PMC4196152; doi:10.18632/oncotarget.1955)
Supplement: Supplementary file 2 [file oncotarget-05-6633-s002.pdf]

ADAM10 mediates trastuzumab resistance and is correlated with survival in HER2 positive breast cancer

Supplemental Information

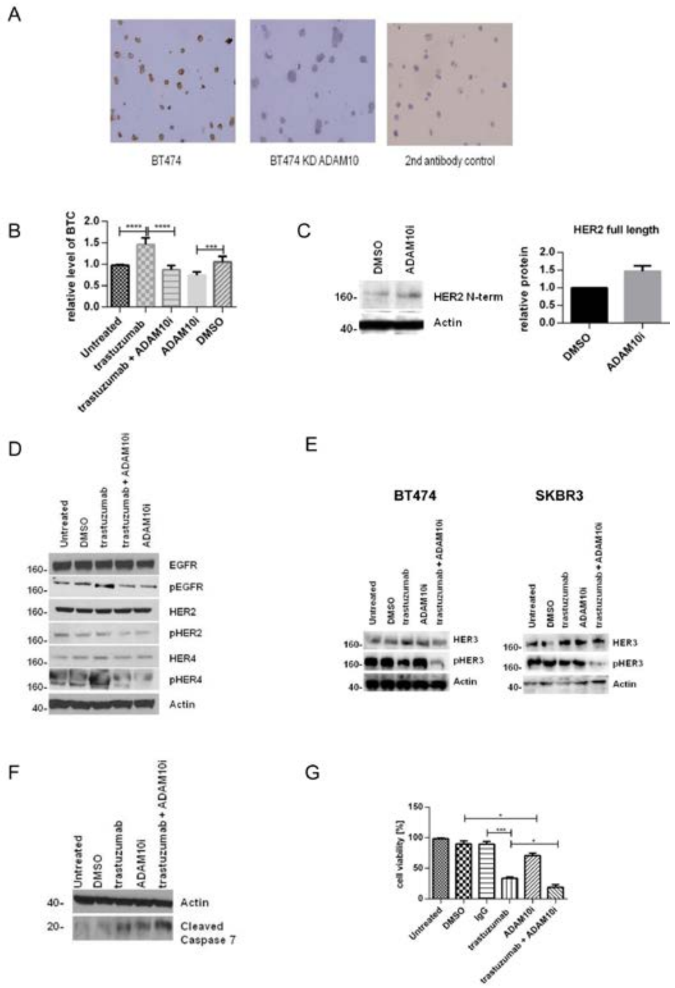

Figure S1: ADAM10 inhibition decreases trastuzumab induced activation of HER receptors and inhibits cell viability. (A) Antibody verification for IHC staining. BT474 cell pellets were generated from parental cells or from cells transfected with siADAM10 for 72h (middle panel). Cells were stained for ADAM10 expression and a second antibody control is shown (right panel). (B) BT474 cells were treated with 40µg/ml trastuzumab, 5µM ADAM10 inhibitor INCB8765 (ADAM10i), or their combination in serum-free media as indicated for 24h. Betacellulin levels in the media were assessed in triplicate using ELISA. (C) SKBR3 cells were treated with 5µM ADAM10 inhibitor INCB8765 (ADAM10i) or control in serum-free media as indicated for 24h and cell lysates were subjected to western blot using an antibody directed to the extracellular domain of HER2. A semi-quantification of three blots is shown. (D) BT474 cells were treated with 40µg/ml trastuzumab, 5µM ADAM10 inhibitor

INCB8765 (ADAM10i), or their combination in serum-free media as indicated for 24h and cell lysates were subjected to western blot. A semi-quantification of three blots is shown. (E) BT474 and SKBR3 cells were treated with 40µg/ml trastuzumab, 5µM ADAM10 inhibitor INCB8765 (ADAM10i), or their combination in serum-free media as indicated for 24h and cell lysates were subjected to western blot. (F) SKBR3 cells were treated with 40µg/ml of trastuzumab, 5µM ADAM10 inhibitor INCB8765 (ADAM10i), or their combination in serum-reduced media for 72h and the expression of cleaved caspase 7 was assessed by western blot. (G) For MTT assays BT474 cells were seeded in triplicate and treated with 40µg/ml trastuzumab, 5µM ADAM10 inhibitor INCB8765 (ADAM10i), or their combination in serum-reduced media for 5 days before analysis. Graphs show means ± SD. Statistical significance was calculated using one-way ANOVA, and Bonferroni's multiple comparison was used to compare indicated groups. P values are shown (*$p \leq 0.05$, **$p \leq 0.01$, ***$p \leq 0.001$, ****$p \leq 0.0001$).

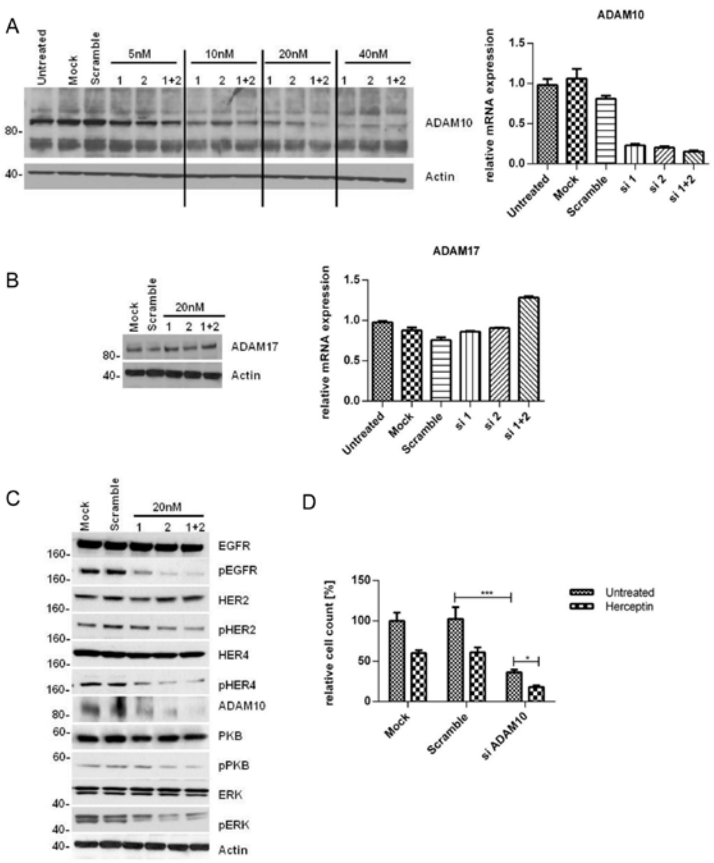

Figure S2: The optimization of ADAM10 knockdown; ADAM10 knockdown inhibits activation of HER receptors and decreases cell viability. (A) Optimization of ADAM10 knockdown. BT474 cells were transfected with different concentrations of two siRNAs or their combination. Western blot analysis was performed after 72h and mRNA levels were assessed by qRT-PCR in triplicate after 48h. (B) To exclude off-target effects, BT474 cells were transfected with 20nM of siRNA against ADAM10 before the protein levels of ADAM17 were assessed by western blot after 72h and mRNA levels were determined 48h post transfection in triplicate by qRT-PCR. (C) ADAM10 was knocked down in BT474 cells using 20nM of siRNA and protein levels were assessed after 72h by western blot. A semi-quantification of three blots is shown. (D) In cell counting assays, BT474 cells transfected with 20nM of siRNA against ADAM10 were seeded in triplicate and treated the next day with 40µg/ml trastuzumab as indicated for 5 days. Graphs show means ± SD. Statistical significance was calculated using one-way ANOVA, and Bonferroni's multiple comparison was used to compare indicated groups. P values are shown (*p ≤ 0.05, **p ≤ 0.01, ***p ≤ 0.001, ****p ≤ 0.0001).

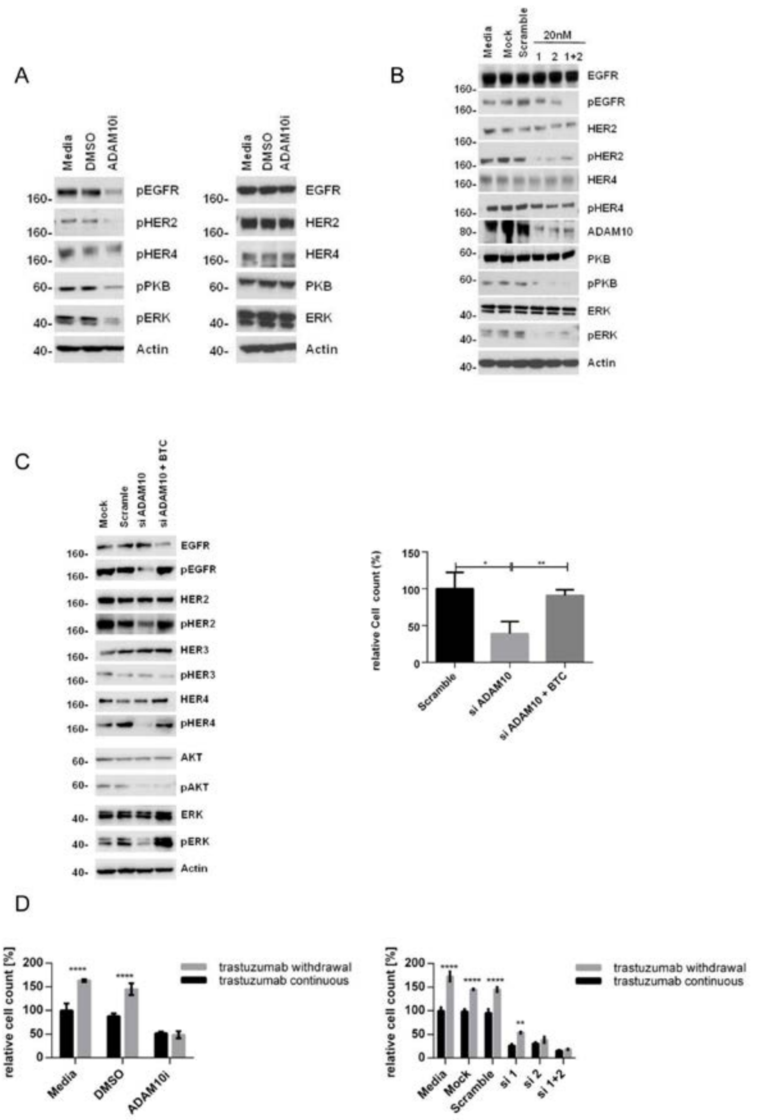

Figure S3: ADAM10 inhibition or knockdown decreases activation of HER receptors and cell viability in trastuzumab resistant cell lines. All resistant cells were continuously treated with 40µg/ml trastuzumab in the experiments below unless otherwise stated. (A) BT474 resistant cells were treated with 5µM ADAM10 inhibitor INCB8765 (ADAM10i) for 24h in serum-free media or (B) BT474 resistant cells were transfected with 20nM of siRNA against ADAM10 for 72h. Quantification of three independent experiments is shown. The relative protein levels from the semi-quantification of three western blots are shown. (C) ADAM10 was knocked down and the cells were co-stimulated with 50ng/ml betacellulin as indicated for 5 days before cell counting experiments and for 72h before western blot analysis. (D) For cell counting studies, trastuzumab was withdrawn from BT474 resistant cell overnight. The next day one group was re-treated ("continuous") for the duration of the experiment, whereas the "withdrawal" group remained without trastuzumab treatment. Cells were

treated in triplicate with 5µM ADAM10 inhibitor INCB8765 (ADAM10i) in serum-reduced media or cells were transfected with 20nM of siRNA against ADAM10 as indicated for 5 days. Graphs represent data from three independent experiments and show means ± SD. Statistical significance was calculated using one-way ANOVA, and Bonferroni's multiple comparison was used to compare indicated groups. For (C) two-way ANOVA and Tukey's multiple comparison was used to compare the groups. P values are shown (*$p \leq 0.05$, **$p \leq 0.01$, ***$p \leq 0.001$).

A

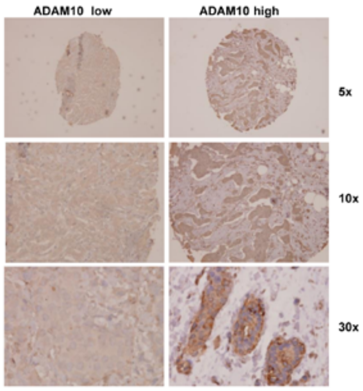

B

Ki 67 [%]					
Basal ADAM10	Basal	21 days trastuzumab	Ratio post/pre trastuzumab	after neoadjuvant therapy	Ratio post/pre neoadjuvant
high	28	25	0.89	5	0.18
	28	30	1.07	40	1.43
	23	30	1.30	33	1.43
	35	35	1.00	65	1.86
low	50	50	1.00	18	0.36
	28	20	0.71	25	0.89
	80	60	0.75	50	0.63
	40	35	0.88	40	1.00
	13	7	0.54	5	0.38
	30	18	0.60	11	0.37

Tumor size [mm]					
Basal ADAM10	Basal	21 days trastuzumab	Ratio post/pre trastuzumab	after neoadjuvant therapy	Ratio post/pre neoadjuvant
high	40	45	1.13	25	0.63
	50	70	1.40	20	0.40
	40	55	1.38	17	0.43
	80	60	0.75	17	0.21
low	50	50	1.00	15	0.30
	60	40	0.67	21	0.35
	35	26	0.74	15	0.43
	25	15	0.60	14	0.56
	40	20	0.50	10	0.25
	45	38	0.84	7	0.16

Figure S4: Examples of ADAM10 IHC staining and correlation of ADAM10 staining with tumor response of patients. (A) Staining for basal ADAM10 expression by IHC; two samples are shown. (B) Values for post/pre-treatment ratios of Ki67 and tumor sizes of patients receiving one dose of trastuzumab (8mg/kg for 21 days) followed by standard treatment (trastuzumab 6mg/kg q21 and docetaxel 100mg/kg q21) are shown.

Table S1: The patient characteristics and the tumor data from HER2 positive breast cancer patients undergoing neoadjuvant chemotherapy and trastuzumab. HER2 positive breast cancer patients were given one dose of trastuzumab (8mg/kg) followed by 4 cycles of neoadjuvant docetaxel chemotherapy 100 mg/m^2 with 6mg/kg trastuzumab q21 before surgery were assessed. Patients' characteristics and the tumor data were stratified in relation to basal (= pre-treatment) ADAM10 expression and the differences between the two groups were assessed using Fisher's exact test. P-values are shown.

Features	Total Number	ADAM10 expression		P-Value
		High	Low	
**Tumor size baseline**				0.6
Smaller than 2cm	0	0	0	
2-5cm	4	3 (50%)	1 (25%)	
≥ 5cm	6	3 (50%)	3 (75%)	
**Nodal status**				1.0
Negative	5	3 (50%)	2 (50%)	
Positive	4	3 (50%)	1 (25%)	
Unknown	1		1 (25%)	
**ER status**				0.6
Negative	6	3 (50%)	3 (75%)	
Positive	4	3 (50%)	1 (25%)	
**Grade**				1.0
3	8	5 (83%)	3 (75%)	
1-2	2	1 (17%)	1(25%)	
**Age**				0.2
< 55	4	1 (17%)	3 (75%)	
≥ 55	6	5 (83%)	1 (25%)	

Table S2: The patient characteristics and the tumor data from a cohort of HER2 positive breast cancer patients. Tissue microarrays (TMAs) from HER2 positive breast cancer patients in Oxford were stained for basal ADAM10 expression by IHC. Patients' characteristics and tumor data was stratified in relation to ADAM10 expression and expression and the differences between the two groups were assessed using Fisher's exact test. P-values are shown.

**Features**	**Total Number**	**ADAM10 expression**		**P-Value**
		**High**	**Low**	
**Tumour size**				0.8
Smaller than 2cm	22	7 (37%)	15 (30%)	
2-5cm	40	10 (53%)	30 (60%)	
≥ 5cm	7	2 (10%)	5 (10%)	
**Nodal status**				1.0
Negative	35	9 (47%)	26 (52%)	
Positive	32	9 (47%)	23 (46%)	
Unknown	2	1 (5%)	1 (2%)	
**ER status**				0.8
Negative	18	4 (21%)	14 (28%)	
Positive	50	14 (74%)	36 (72%)	
Unknown	1	1 (5%)		
**Grade**				1.0
3	51	14 (74%)	37 (74%)	
1-2	18	5 (26%)	13 (26%)	
**Age**				0.6
< 55	21	7 (37%)	14 (28%)	
≥ 55	47	12 (63%)	36 (72%)	
**Relapse**	10	6	4	
**Death**	5	2	3	

Table S3: Multivariate analysis of ADAM10 expression in relation to known clinical co-variates in HER2 positive breast cancer patients. Multivariate analysis of ADAM10 in (A-D) RFS and (E-H) OS with known clinical factors: age, grade, node status, ER status, and size. (A) and (E) multivariate analysis for RFS and OS considering all factors. (B) and (F) multivariate analysis without grade. (C) and (G) multivariate analysis without node status. (D) and (H) multivariate analysis without grade or node status. (I) Co-variants correlation.

A

Multivariate analysis for RFS					
	coef	exp(coef)	se(coef)	z	p
ADAM10	2.387	1.09E+01	7.87E-01	3.03218	0.0024
Age	1.396	4.04E+00	9.02E-01	1.54819	0.12
Grade	18.629	1.23E+08	1.26E+04	0.00148	1
Nodes	-1.455	2.33E-01	9.60E-01	-1.5162	0.13
ER	0.853	2.35E+00	7.23E-01	1.18014	0.24
factor(Size)2 (≥2 and < 5)	-0.25	7.79E-01	1.30E+00	-0.19249	0.85
factor(Size)3 (≥5)	1.882	6.57E+00	1.36E+00	1.37948	0.17
Likelihood ratio test=23.5 on 7 df, p=0.00141, n=66, number of events= 10, (3 observations deleted due to missingness)					

B

Multivariate analysis for RFS w/o Grade					
	coef	exp(coef)	se(coef)	z	p
ADAM10	2.437	11.438	0.778	3.1339	0.0017
Age	1.2533	3.502	0.87	1.4399	0.15
Nodes	-1.4834	0.227	0.978	-1.5169	0.13
ER	1.0586	2.882	0.706	1.4997	0.13
factor(Size)2 (≥2 and < 5)	0.0601	1.062	1.262	0.0476	0.96
factor(Size)3 (≥5)	2.2487	9.475	1.387	1.6214	0.1
Likelihood ratio test=22.2 on 6 df, p=0.00112, n=66, number of events= 10, (3 observations deleted due to missingness)					

C

Multivariate analysis for RFS w/o Node status					
	coef	exp(coef)	se(coef)	z	p
ADAM10	2.219	9.20E+00	7.68E-01	2.89	0.0039
Age	1.41	4.10E+00	8.13E-01	1.73523	0.083
Grade	18.93	1.66E+08	1.06E+04	0.00179	1
ER	1.107	3.02E+00	7.21E-01	1.53602	0.12
factor(Size)2 (≥2 and < 5)	0.345	1.41E+00	1.21E+00	0.28509	0.78
factor(Size)3 (≥5)	2.907	1.83E+01	1.29E+00	2.25321	0.024
Likelihood ratio test=21.4 on 6 df, p=0.00157, n=68, number of events= 10, (1 observation deleted due to missingness)					

D

Multivariate analysis for RFS w/o Grade and Node status					
	coef	exp(coef)	se(coef)	z	p
ADAM10	2.25	9.48	0.758	2.97	0.003
Age	1.2	3.32	0.764	1.57	0.12
ER	1.37	3.92	0.701	1.95	0.051
factor(Size)2 (≥2 and < 5)	1.1	2.99	1.095	1	0.32
factor(Size)3 (≥5)	3.7	40.3	1.256	2.94	0.0033
Likelihood ratio test=19.4 on 5 df, p=0.0016, n=68, number of events= 10, (1 observation deleted due to missingness)					
